# Effect of internal structure and resin deformability on drying rate and stress in convective drying of silica–latex coatings

**DOI:** 10.1140/epje/s10189-024-00432-9

**Published:** 2024-07-04

**Authors:** Hiroaki Tanaka, Yoshiyuki Komoda, Takafumi Horie, Naoto Ohmura

**Affiliations:** 1 Department of Chemical Science and Engineering, Kobe University, 1-1, Rokkodai-Cho, Nada-ku, Kobe, Hyogo 657-8501 Japan; 2 Department of Chemical Engineering, Osaka Metropolitan University, 1-1 Gakuen-Cho, Nakaku, Sakai, Osaka 599-8531 Japan

## Abstract

Latex paint is an aqueous dispersion of nano-sized polymer particles that can form a thin film by itself or mixed with rigid particles. We have developed an apparatus that can simultaneously measure drying rate and stress generation and have investigated the film formation process of a latex-only coating layer under convection drying. In the present study, we adopted the same method to investigate the film formation process of the silica–latex coating layer. As a result, we were able to systematically correlate the drying rate change by the equivalent thickness of latex particles accumulated with silica particles at the drying surface. Furthermore, it is unveiled that the drying rate in the former stage depends on drying temperature, while the drying rate changed to be dominated by silica content after the particle-packing layer was formed over the entire coating layer. On the other hand, the model we proposed for stress generation, considering the temperature effect on latex deformability, was found to be applicable to the present experimental system by replacing a portion of deformable particles with rigid particles.

**Graphical abstract:**

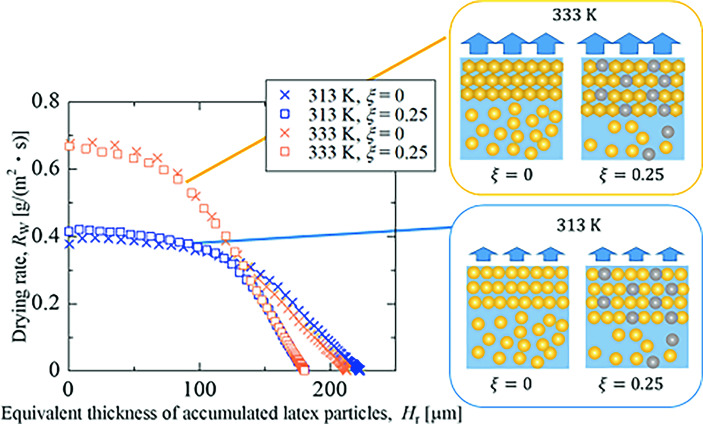

## Introduction

Latex paints are aqueous dispersions of nano-sized polymer particles and attract attention to reduce organic solvent usage compared to solvent-based ones. The polymer particles have the character to deform and coalesce, producing a continuous film. Understanding the film formation process is essential to further develop this environmentally waterborne coating. Therefore, the film formation process of latex coatings has been studied for many decades [1–7]. The drying process of latex coating is generally described in three stages. Water evaporation concentrates the latex dispersion locally at the top of the coating layer (Stage 1). As the drying progresses, the packing structure of polymer particles is developed downward, and the entire coating layer changes to the packing structure (Stage 2). Finally, the deformation and coalescence of polymer particles form a continuous polymer film (Stage 3).

In the drying process of latex coating, the deformation mechanism of polymer particles is affected by various factors. Dillon et al. [1] reported that the coalescence of polymer particles is due to the surface tension between the particles and air, called dry sintering. On the other hand, Brown [2] proposed that the coalescence of polymer particles occurs by capillary pressure from the air–water surface tension when the closest packed structure of the particles is formed in the layer. He also noted that polymer-air and polymer-water surface tension might contribute to deformation. When polymer particles are easily deformed at a high drying temperature, deformed polymer particles form the packing structure at the top, which is known as wet sintering, and suppress the evaporation of water. Later, Routh and Russel [8, 9] presented a comprehensive model for film formation by using the dimensionless numbers, $\overline{\lambda }$ and $Pe$.

In the drying process of particle dispersion coating layers, the non-uniform distribution of non-sedimentary particles is explained by the Péclet number, $Pe$ [10–14]. $Pe$ is the ratio of the evaporation rate to the diffusion rate of dispersed particles. The diffusion coefficient of particles due to their Brownian motion in a liquid with a viscosity $\mu$ is given by the Stokes–Einstein equation (Eq. (1)).

1 $$D=\frac{kT}{6\pi \mu r}$$

where $r$ is the radius of the polymer particle, $k$ is the Boltzmann constant, and $T$ is the temperature. Then, the Péclet number is expressed by Eq. (2) using the initial evaporation rate, $v,$ and the initial thickness of the coating layer, ${H}_{0}$.

2 $$Pe=\frac{{H}_{0}v}{D}=\frac{{H}_{0}v\cdot 6\pi \mu r}{kT}$$

**Fig. 1:**
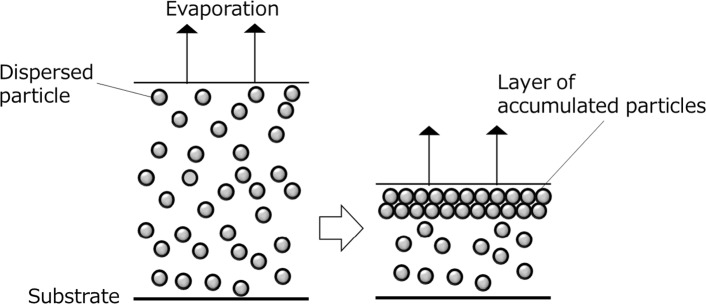
Schematic diagram of particle distribution changes during drying (Pe>> 1)

In the drying process of latex coatings above room temperature, the Péclet number is usually much larger than 1, and latex particles accumulate primarily under the drying surface (Fig. 1).

Routh and Russel [8] defined a dimensionless parameter $\overline{\lambda }$ to classify the deformation mechanism of polymer particles, which is the ratio of the evaporation time to the deformation time; then, $\overline{\lambda }$ is defined by Eq. (3).

3 $$\overline{\lambda }=\frac{{r\eta }_{0}/{\gamma }_{\text{wa}}}{{H}_{0}/v}=\frac{vr{\eta }_{0}}{{\gamma }_{\text{wa}}{H}_{0}}$$

The characteristic time for particle deformation by viscous flow is proportional to its zero-shear rate viscosity, ${\eta }_{0}$, which resists deformation and is inversely related to the relevant interfacial tension, ${\gamma }_{\text{wa}}$, which drives deformation. The initial thickness is divided by the initial evaporation rate to calculate the characteristic time for drying. They predict that the deformation occurs by wet sintering for $\overline{\lambda }<1$, capillary deformation for $1<\overline{\lambda }<{10}^{2}$, and dry sintering for ${10}^{4}<\overline{\lambda }$. Many studies on latex coatings focusing on the deformability of polymer particles have been reported, for example, Gonzalez et al*.* systematically investigated the relationship between infrared drying conditions and the deformation mechanism proposed by Routh and Russel [15]

The stress development of latex coating during drying is also a major topic [16–19]. The latex coating layer can relax the stress generated during drying due to the deformation of polymer particles and produce a uniform, crack-free thin film. Although stress development relates to the internal structure change of the coating and the deformation of polymer particles with the evaporation of water, many experimental studies measured the stress arising in the coating layer as a function of time, not a function of water content, which cannot reveal the relationship between stress and internal structure. Our previous work measured the variation of moisture content of latex coatings in convective drying from the coating temperature change and discussed the effect of drying temperature on the film formation processes of latex coating [20]. We further installed equipment to measure the deflection of the coating layer and successfully conducted the simultaneous measurement of the drying rate and stress of latex coating [21]. The experimental setup used in the study requires neither a precise electrical balance for water loss measurements nor accurate optics for stress measurements. Thus, our system is suitable for convective drying with hot air flow and accurately measures moisture content change even in the falling drying rate period, where the stress usually exhibits a significant increase.

Latex paints are sometimes used solely but are often mixed with particles to impart various functionalities to the resultant thin polymer film. On the contrary, a small portion of latex paint is mixed with relatively dense particle dispersion. After drying, the coating layer of such a particle dispersion produces a thin layer in which particles are connected via polymer. In this case, latex paint is usually called a binder, and polymer particles play a role in binding particles and reducing crack formation. In particular, many studies have been conducted on the drying of mixtures of rigid spheres and latex dispersions to understand crack suppression and stress generation/relaxation behavior [22–24]. Martinez et al*.* reported that latex coatings containing 40 vol% or more silica particles can reduce drying time, but stress exhibited a significant increase and decrease [25]. The drying temperature changes the effect of rigid particle addition on the film formation process and stress development, as well as the deformability of polymer particles. However, the effect of drying temperature on the film formation process of latex paints including rigid spheres has yet to be systematically studied.

As for the latex paint used in our previous studies, it was revealed that the film formation process and the corresponding model were changed with the deformation mechanism of polymer particles: capillary deformation at 313 K and wet sintering at 333 and 353 K [20]. Although higher drying temperature increases drying rate and polymer deformability, the effect of the coexistence of rigid spheres on the film formation process has not been clarified. The present study studies the film formation process of latex coating containing rigid spheres at a fraction less than 25vol% of non-volatile components, where rigid spheres are dispersed in the continuous polymer phase in the dried film. Using our original experimental setup for simultaneous measurement of the moisture content and stress development, the effects of rigid sphere content and drying temperature on the film formation process have been discussed. Since particle size significantly changes the Brownian motion and affects the distribution in the drying layer, we selected the rigid spheres that have a similar size to the polymer particles to neglect the size effect on the particle packing process. The objective of this study is to clarify how the dispersed rigid spheres affect the packing process of polymer particles and how our proposed model for latex paint needs to be modified with the coexistence of rigid spheres.

## Experiment

### Sample

To investigate the effect of the content of rigid particles on the drying process of the mixture of latex dispersion and spheres, we first prepare dispersions of latex particles and rigid spheres having the same solid content.

An aqueous dispersion of acrylic latex (50 wt%, specific density 1.17, glass transition temperature, ${T}_{\text{g}}=288\text{ K}$, particle radius, $r=110\text{ nm}$, Japan Coating Resin Corp.) is diluted with de-ionized water to a solids volume fraction of 0.40. On the contrary, silica particles (specific density 1.90, particle radius, $r=250\text{ nm}$, NIPPON SHOKUBAI Corp.) were used as rigid particles and dispersed in de-ionized water at a solid volume fraction of 0.40. The pH of these dispersions is adjusted to 9.0 by adding an aqueous solution of sodium hydroxide to prevent aggregation.

These dispersions were mixed to prepare a silica–latex dispersion with a constant volume fraction of non-volatile components of 40vol%. The silica content $\xi$ is defined by the following equation as the volume fraction of silica particles to non-volatile components (silica and latex) using the volumes of silica ${V}_{\text{s}}$ and latex ${V}_{\text{r}}$. The initial volume fraction of the non-volatile components was constant at 40vol%

4 $$\xi =\frac{{V}_{\text{s}}}{{V}_{\text{s}}+{V}_{\text{r}}}$$

In general, as the particle content in the coating film increases, the coating layer becomes more susceptible to producing defects. Martinez et al*.* [25] investigated stress changes in a coating layer of the mixture of silica particles (570 nm) and latex particles (195–390 nm) and noted that defects appeared after drying in the coating layer with a thickness of 300 µm at the silica content more than 40vol%. Therefore, in the present, we prepared the silica–latex dispersion with silica volume fraction $\xi$ = 0 to 0.25, which are expected to form a uniform coating film without defects.

The prepared silica–latex dispersion was applied on a copper substrate by a blade coating technique. The copper substrate has a width of 1.5 cm, a length of 10 cm, and a thickness of 0.3 mm. The coating area is 7.5 cm long and 1.5 cm wide. The weight and thickness of the pre-drying coating layer were calculated from the mass difference before and after drying and the initial composition.

### Apparatus and method for drying experiment

**Fig. 2:**
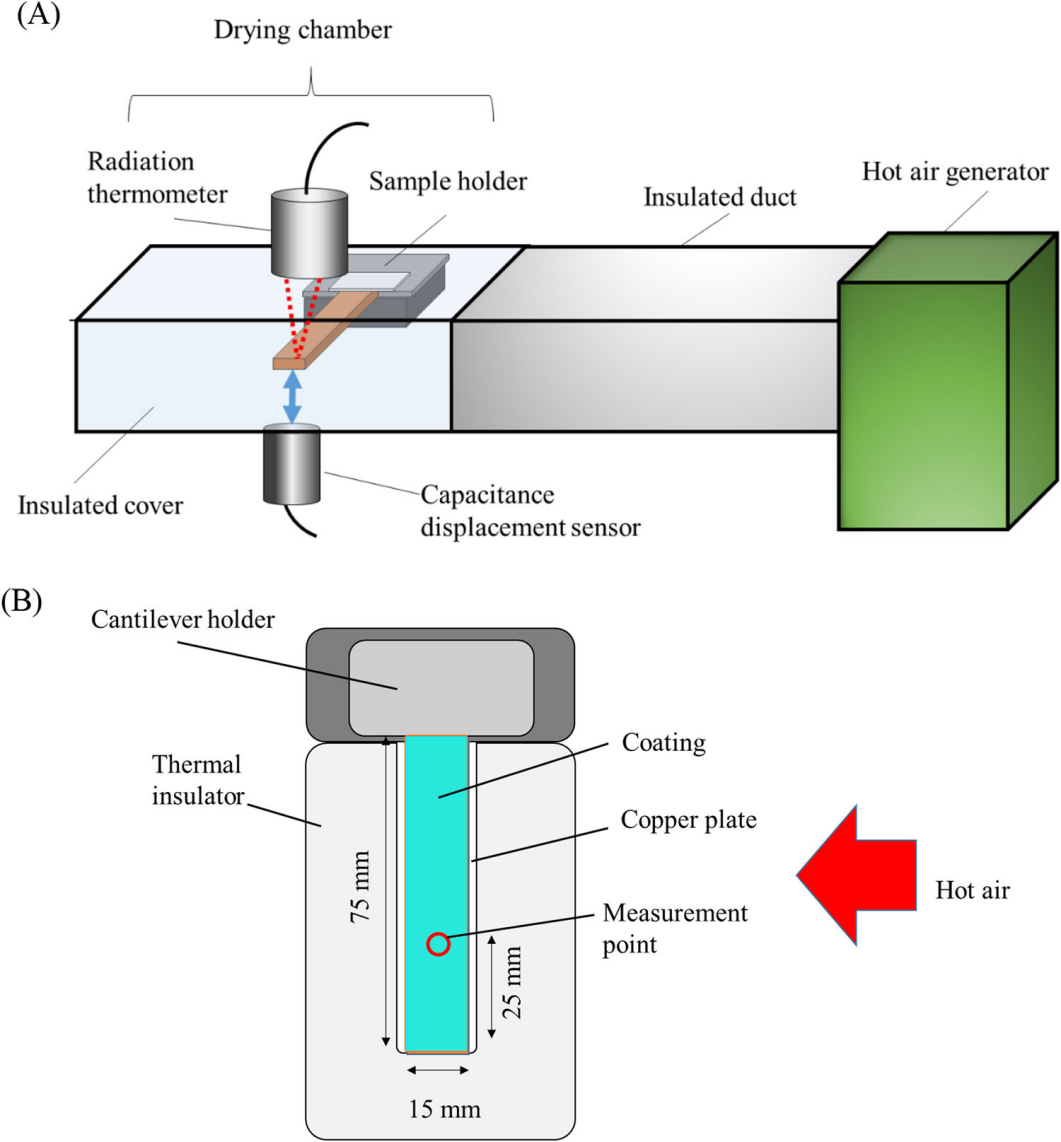
Experimental setup for simultaneous measurement of drying rate and stress. **A** is a schematic diagram of the entire experimental setup, and **B** is a schematic diagram of the inside of the chamber

In the present study, we have simultaneously measured the drying rate and drying stress that arose in the coating layer under convective drying conditions. Figure 2 illustrates the experimental apparatus, identical to the apparatus used in the previous studies. The sample-coated copper substrate was placed in an insulated drying chamber connected to a hot air generator (HAS-11, Kansai Electric Heat Corp.). The temperature, relative humidity, and flow rate of the hot air are monitored by using an anemometer (CLIMOMASTER 6501–00, KANOMAX JAPAN INC.). The hot air temperature was at 313 or 333 K, and the relative humidity and flow rate were roughly constant at 50% RH and 1.8 m/s during the drying experiment. We measured the sample temperature using a radiation thermometer (CTL-CF1-C3 MICRO-EPSILON) installed at the top wall of the chamber to calculate the time variation of the water content of the coating layer. The detail of the calculation of water content is found in Sect. 2.3. We also measured the deflection of the substrate using a capacitance displacement sensor (SMAV-SEN-1, Syouei System Corp.) fixed below the substrate to measure the stress that arose in the drying coating layer. The deflection can be converted to stress using the Corcoran equation, [26] which will be described in Sect. 2.4. In the present study, to minimize the edge effect on the drying rate measurement, the thermometer and displacement sensor are fixed so that the measuring areas are located in the middle of the coating layer. They are connected to a PC to continuously measure the temperature and deflection at the time interval of 0.05 s. The details of the equipment can be found elsewhere [21].

### Measurement of water content

The temperature change method [27, 28] calculates the amount of residual water in the coating layer from the energy balance and mass balance equations. The heat from the outside is consumed to evaporate water in the coating layer or raise the temperatures of the coating layer and substrate. Therefore, an instantaneous mass change of water, ${\Delta W}_{\text{W}}$, within a time interval, $\Delta t$, is expressed by Eq. (5).

5 $$-{\Delta W}_{\text{w}}=\frac{Ah\left({T}_{\text{a}}-{T}_{\text{m}}\right)\Delta t-\left({W}_{\text{w}}{C}_{\text{w}}+{W}_{\text{r}}{C}_{\text{r}}+{W}_{\text{s}}{C}_{\text{s}}+{W}_{\text{b}}{C}_{\text{b}}\right)\Delta {T}_{\text{m}}}{{L}_{\text{w}}}$$

where *C* and *W* represent the specific heat capacity and mass, respectively. The subscriptions w, r, s, and b indicate water, resin, silica, and substrate. $t$ is the drying time, and $A$ is the evaporation area. ${T}_{\text{a}}$ is the hot air temperature, and ${T}_{\text{m}}$ is the temperature of the coating layer or substrate, measured by the radiation thermometer. $h$ is the boundary heat transfer coefficient at the surface of the coating layer. The boundary heat coefficient *h* was determined to satisfy the heat and mass balance equations to calculate moisture content from the sample temperature. Constant *h* under the same hot air temperature means that the convection drying conditions were stable for all experiments.

### Measurement of drying stress

The deflection $\Delta d$ was converted to drying stress, $\sigma$ using the Corcoran equation.

6 $$\sigma =\frac{\Delta d{E}_{\text{b}}{{H}_{\text{b}}}^{3}}{3{H}_{\text{c}}{l}^{2}\left({H}_{\text{b}}+{H}_{\text{c}}\right)\left(1-{\nu }_{\text{b}}\right)}+\frac{\Delta d{E}_{\text{c}}\left({H}_{\text{b}}+{H}_{\text{c}}\right)}{{l}^{2}\left(1-{\nu }_{\text{c}}\right)}$$

where *E*, *H*, *l* and *ν* represent Young's modulus, thickness, length for measuring the substrate deflection, and Poisson's ratio. The subscripts c and b indicate the variables for the coating and substrate, respectively. The copper substrates used have a thickness of 300 μm and the following properties: *E*_b_ = 118 GPa, *l* = 75 mm, and *ν*_b_ = 0.33. The modulus of the coating is much smaller than the substrate modulus, so the second term was neglected. This technique cannot measure stress distribution throughout the coating layer due to local microstructural differences. Hence, the results should be considered the average stress of the entire coating layer.

## Result & discussion

### Drying rate of silica–latex coating

Our previous study dealt with the drying process of the latex-only coating layer in a chamber supplied with various hot air temperatures [20]. We concluded that the deformation mechanism of polymer particles is “capillary deformation” at 313 K or “wet sintering” at 333 K or higher. Here, the former part of the present study discusses the effect of silica particles on the drying rate and the behavior of the packing structure of polymer particles exhibiting different deformation mechanisms.

**Fig. 3:**
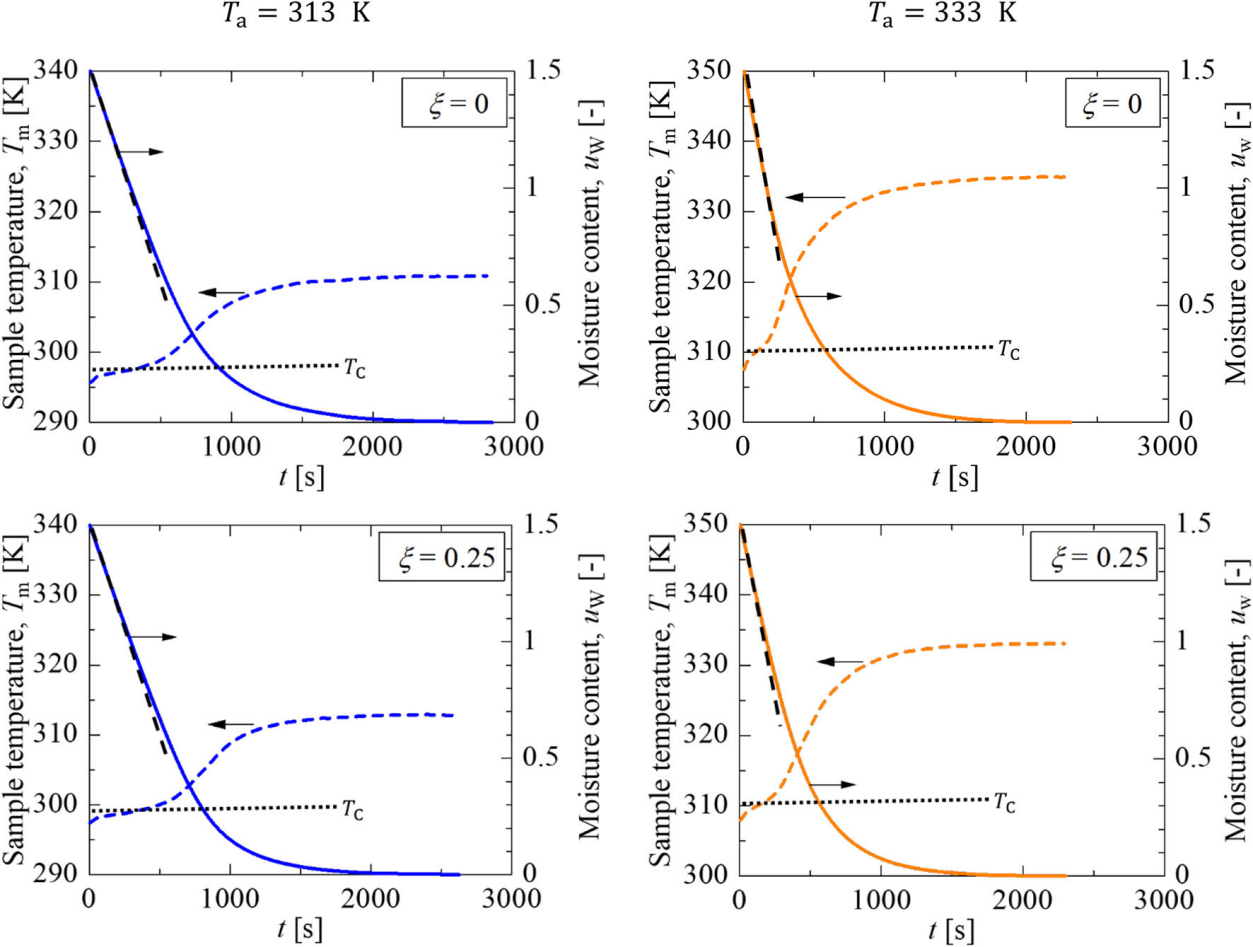
Histories of the sample temperature and moisture content of latex-silica coating as a function of drying time for different silica volume fractions and drying temperatures

Figure 3 shows the variation in the sample temperature, ${T}_{\text{m}}$, and the moisture content, ${u}_{\text{W}},$ calculated from the sample temperature change at silica volume fractions of *ξ* = 0, 0.25 at a drying temperature of 313 K and 333 K. Equation (7) gives the moisture content of the silica–latex coating, ${u}_{\text{W}}$, the volume ratio of water to the non-volatile components.

7 $${u}_{\text{W}}=\frac{{V}_{\text{w}}}{{V}_{\text{s}}+{V}_{\text{r}}}=\frac{1}{{\phi }_{\text{NV}}\left(t\right)}-1$$

where ${V}_{\text{w}}$ is the volume of water. Since the initial volume fraction of the non-volatile components was 40vol%, the initial moisture content ${u}_{\text{w}0}$ was 1.5 for all silica volume fractions.

For any silica content, the sample temperature rose slowly just after the start of drying and took on a roughly constant value,${T}_{\text{C}}$, which corresponds to the constant drying rate period with a liner decrease in the moisture content as indicated by the black dashed lines. The decreasing slope, which is the initial evaporation rate,${v}_{0}$, was 0.40 (Ta = 313 K) or 0.66 μm/s (Ta = 333 K), regardless of silica content. After the constant drying rate period, the sample temperature shows a drastic increase, corresponding to the transition to the falling drying rate period. The moisture content at the end of the constant drying rate period and the decreasing trend during the falling drying rate period, which must be caused by the difference in the internal structure change, vary with the experimental conditions. Finally, the sample temperature reaches the hot air temperature, indicating the end of drying. Since the sample temperature finally becomes higher than the glass transition temperature of the latex polymer (288 K), polymer particles are finally fused to form uniform films.

Table 1 summarized the experimental parameters. Péclet numbers for silica and polymer particles at 313 K, calculated using the initial film thickness, initial evaporation rate, and particle size, were much larger than unity. The Péclet number indicates that the packing structure of any particles is formed beneath the drying surface, and a dispersed phase remains below the packing structure during drying. Since the dimensionless parameter $\overline{\lambda }$ was approximately $3\times {10}^{1}$ and is in the range of 10^0^ < $\overline{\lambda }$  < 10^2^, we confirmed that the latex particles are deformed by capillary deformation. Péclet numbers for silica and polymer particles at 333 K were much larger than unity. The dimensionless parameter $\overline{\lambda }$ was on the order of ${10}^{-1}$ due to the less viscous nature of the polymer, suggesting that wet sintering is the dominant mechanism for latex particle deformation. Therefore, the deformed polymer particles may inhibit the evaporation of water even during the formation of the particle packing structure.

**Table 1:** Parameters of the drying experiments at different silica volume fractions and drying temperatures

$\xi$(–)	*H*_0_ (µm)	*v*_0_ (µm/s)	*h* (W m^−2^ K^−1^)	$Pe_{{\text{r}}}$ (–)	${Pe}_{\text{s }}$(–)	$\overline{\lambda }$(–)
$${T}_{\text{a}}=313 \text{K}$$						
0	558	0.399	69.0	$1.12\times {10}^{2}$	–	$3.44\times {10}^{1}$
0.1	622	0.403	68.9	$1.26\times {10}^{2}$	$5.76\times {10}^{2}$	$3.11\times {10}^{1}$
0.25	603	0.416	70.5	$1.26\times {10}^{2}$	$5.74\times {10}^{2}$	$3.32\times {10}^{1}$
$${T}_{\text{a}}=333 \text{K}$$						
0	532	0.677	66.2	$1.75\times {10}^{2}$	–	$3.87\times {10}^{-1}$
0.1	563	0.658	68.6	$1.81\times {10}^{2}$	$8.21\times {10}^{2}$	$3.55\times {10}^{-1}$
0.25	602	0.666	67.8	$1.95\times {10}^{2}$	$8.86\times {10}^{2}$	$3.37\times {10}^{-1}$

**Fig. 4:**
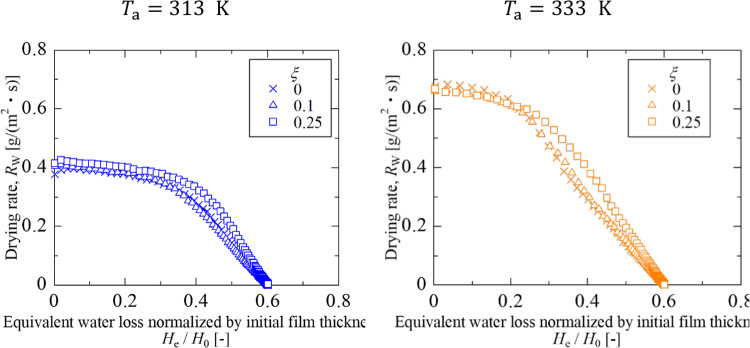
Drying rate as a function of the equivalent thickness to water loss

The effects of drying temperature and silica content are discussed, considering the difference in latex deformation mechanism. While particles are sufficiently dispersed and the surface accumulation layer is sufficiently thin, the drying rate is kept constant and mainly affected by the drying temperature. As particle accumulation progresses, the drying rate is reduced depending on the thickness of the accumulation layer and the deformability of latex particles in the layer. To investigate the effect of particle deformability on the drying rate reduction, Fig. 4 plotted the drying rate as a function of the equivalent thickness of water loss, *H*_e_, which is defined by Eq. (8). To compensate for experimental discrepancies in the coating process, the initial film thickness was used to normalize the equivalent thickness.

8 $${H}_{\text{e}}={H}_{0}{\phi }_{0}\left({u}_{\text{W},0}-{u}_{\text{W}}\left(t\right)\right)={H}_{0}{\phi }_{0}\left(\frac{1}{{\phi }_{\text{NV},0}}-\frac{1}{{\phi }_{\text{NV}}\left(t\right)}\right)$$

For both drying temperatures, it is unveiled that the drying rate rarely changed with the inclusion of 10vol% of rigid spheres. When containing 25vol% silica particle, the drying rate remains higher. The different behavior for $\xi$ = 0.25 can be explained by the percolation theory [29, 30]. In the silica–latex coating, the surface tension of water deforms latex particles but does not silica particles. If silica particles are connected to each other throughout the accumulation layer, the shrinkage of latex particles is significantly suppressed, delaying the onset of the falling drying rate period. Chevalier et al*.* [31] reported that the elastic modulus of the mixture of soft butyl acrylate and rigid polystyrene particles increases sharply when the particle volume fraction approaches 30vol%, where rigid particles are attached to each other to form the packed structure. Therefore, it is reasonable that at a silica content of $\xi$ = 0.25 in this system, the deformation of resin particles was suppressed and the drying rate maintained a high value.

At 333 K, resin deformation accumulated on the surface due to wet sintering can cause resistance to water transport and decrease drying rates. We consider the latex volume within the packed layer to express the magnitude of the resistance to water movement. Assuming that the volume ratio of water loss to the accumulated particles was identical to the initial moisture content, the equivalent thickness of resin particles in the accumulation layer *H*_r_ is estimated by Eq. (9).

9 $${H}_{\text{r}}=\left(1-\xi \right)\frac{{H}_{\text{e}}}{{u}_{\text{W},0}}=\left(1-\xi \right){H}_{0}{\phi }_{\text{NV},0}\left(1-\frac{\frac{1}{{\phi }_{\text{NV}}\left(t\right)}-1}{\frac{1}{{\phi }_{\text{NV}, 0}}-1}\right)$$

**Fig. 5:**
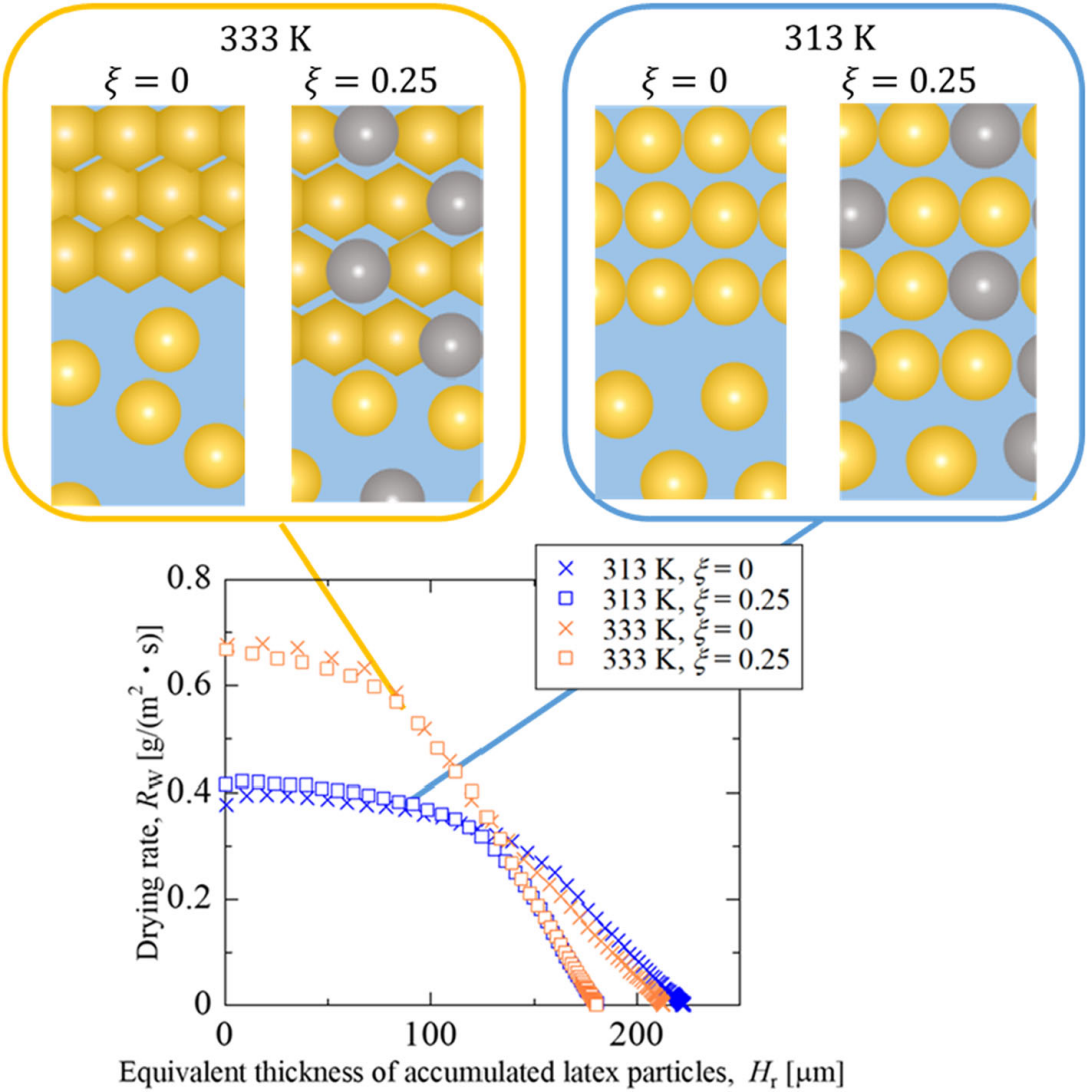
Drying rate as a function of the equivalent thickness of accumulated polymer particles

Figure 5 plots the relationship between the drying rate and equivalent thickness of accumulated resin particles, with and without silica particles at both drying temperatures. It is found that the drying process can be divided into two stages. In the former stage, the drying rate at the same drying temperature exhibited similar behavior regardless of silica inclusion. The drying rates coincide with each other at the equivalent thicknesses of 140 µm, and the drying rate in the latter stage is dominated not by drying temperature but by silica content.

The constant drying rate in the previous stage indicates that the accumulation layer does not inhibit water movement. This suggests that the accumulation layer either has large enough gaps so that the particles do not deform, or that it is thin enough that it does not reduce the rate of water transport. At 333 K, the decrease in drying rate indicates that the deformed resin layer inhibits water movement. The agreement of the drying rate change in the former stage regardless of silica content at each drying temperature reveals that the amount of resin particles in the accumulation layer and its deformability determine the drying rate. This is shown in the schematic diagram of the coating layer in Fig. 5. Furthermore, the coincidence of drying rate at the transition from the former to the latter stage at ${H}_{\text{r}}$ = 140 µm suggests that resin particles form similar structures for all conditions. The volume fraction of resin particles at the moment in the water–latex mixture, ${\phi }_{\text{r}}(t)$, was calculated by Eq. (10) as 0.64 ($\xi$ = 0) or 0.68 ($\xi$ = 0.25), respectively. It is concluded that a random close-packing structure of resin particles is formed at the moment.

10 $${\phi }_{\text{r}}(t)=\frac{{V}_{\text{r}}}{{V}_{\text{w}}(t)+{V}_{\text{r}}}$$

Our previous study proposed the cross-sectional area of water tubes, represented by residual water, dominates the drying rate in the latter stage [15]. Good agreement of the drying rate change in the latter stage means that silica particles do not affect the deformation and coalescence of resin particles in the void space between silica particles.

As a last topic of this section, the effect of silica inclusion on the drying rate in the latter stage will be discussed using the residual moisture content. In the drying process of the coating layer of the silica–latex mixture, silica particles do not contribute to reducing the gap space between latex particles. It is presumed that the ratio of residual water to latex particles determines the drying rate. Therefore, we introduced another moisture content the volume ratio of the residual water to latex particles, as expressed by Eq. (11).

11 $${u}_{\text{w},\text{ r}}=\frac{{V}_{\text{w}}(t)}{{V}_{\text{r}}}=\frac{1-{\phi }_{\text{NV}}\left(t\right)}{\left(1-\xi \right){\phi }_{\text{NV}}\left(t\right)}$$

**Fig. 6:**
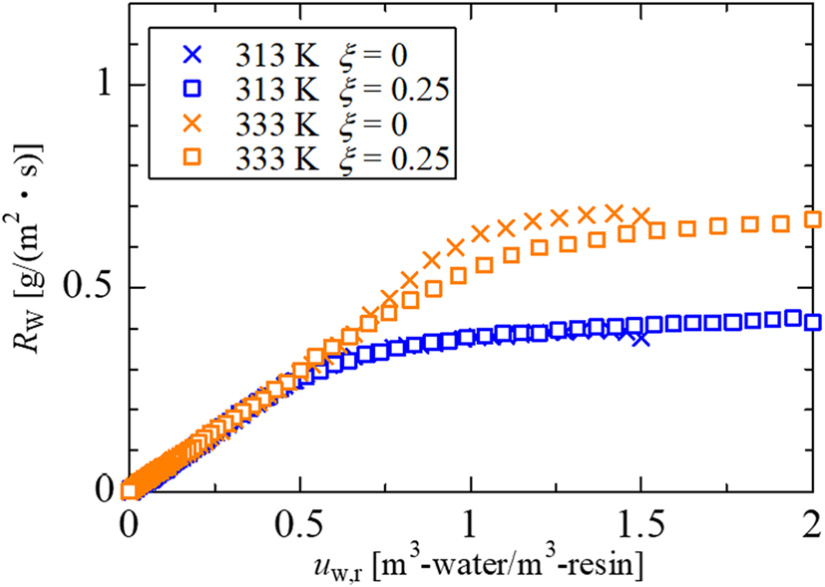
Drying rate curves as a function of latex-based moisture content

Figure 6 shows the relationship between the drying rate and latex-based moisture content for different silica contents at different drying temperatures. The critical equivalent thickness of ${H}_{\text{r}}$ = 140 µm corresponds ${u}_{\text{W},\text{ r}}$ = 0.51 ($\xi$ = 0) and 0.46 ($\xi$ = 0.25), respectively. At the latex-based moisture content less than the critical values, the drying rates show agreement for all experimental conditions. As a result, the drying rate in the latter stage is reasonably explained by the drying process of condensed latex particle dispersion in the space between silica particles.

### Stress development of silica–latex coating

**Fig. 7:**
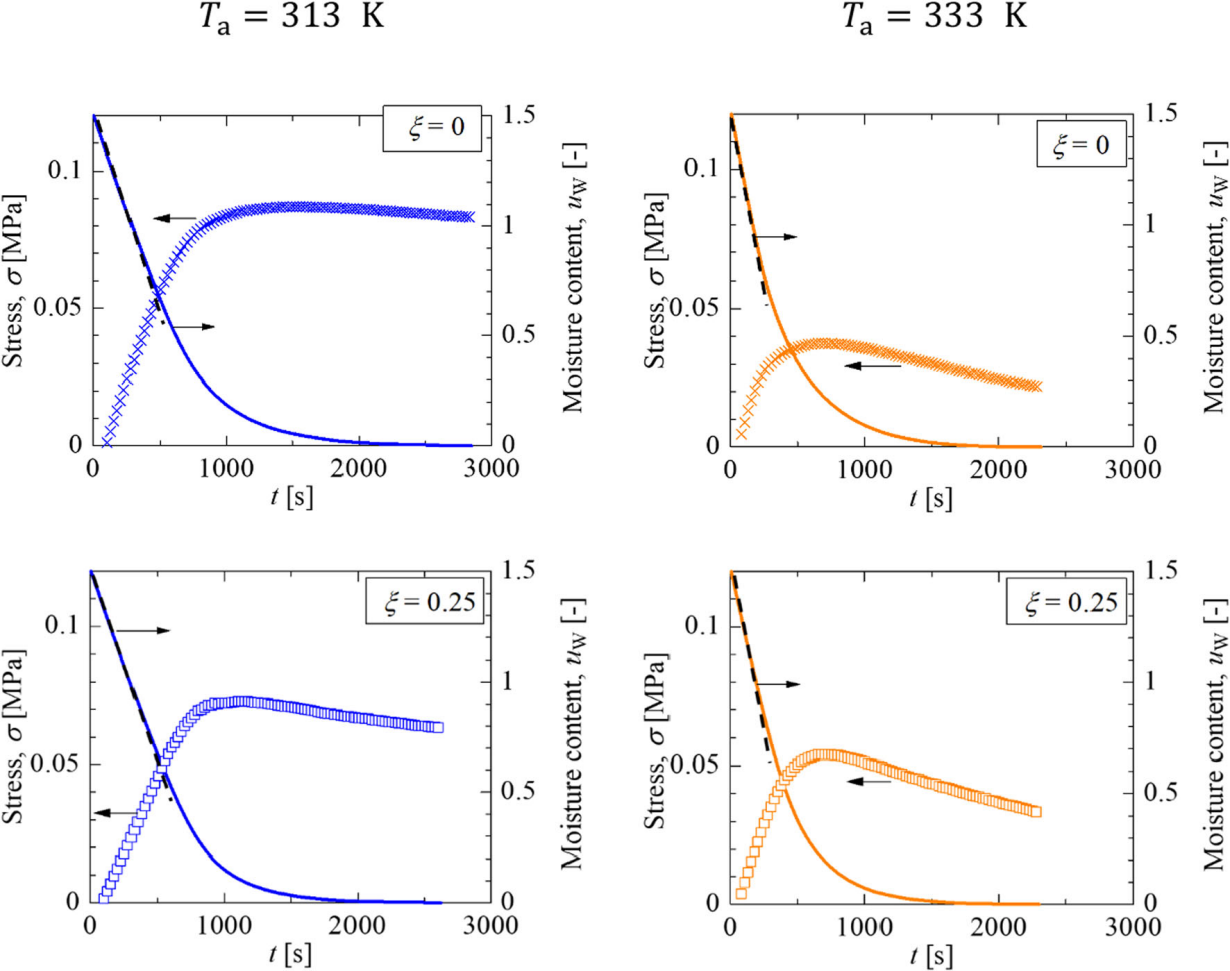
Histories of the stress and moisture content of latex–silica coating as a function of drying time at different silica volume fractions and drying temperatures

This section describes the relationship between the moisture content and stress generated for the latex–silica coating layers at different drying temperatures. The deflection measurement was performed simultaneously with the drying rate measurement described above. Stress development was calculated by the Corcoran equation (Eq. 6) using the deflection. To remove the instability in the early stage of drying, the deflection after 30 s from the start of drying was taken as the zero point. The experimental conditions are found in Table 1. Figure 7 shows the effect of silica inclusion on the changes in the non-volatility-based moisture content and cantilever deflection at a drying temperature of 313 K and 333 K.

At 313 K, the stress increased linearly, while the moisture content decreased at a constant rate. The addition of silica particles did not change the increasing trend of the stress. When the decreasing rate of the moisture content slows down, the increasing behavior of the stress is changed. The stress attains a constant value, indicating stress is stored in the dried coating layer. Toward the end of drying, the network between resin particles begins to deform, and the stress is gradually relaxed.

At 333 K, the stress increased linearly in the constant drying rate period and gradually decreased in the falling drying rate period regardless of silica content. These trends are the same as the characteristics of stress development at 313 K. However, the maximum value of the developed stress is sufficiently small, and stress relaxation occurs within a short time from the start of drying.

**Fig. 8:**
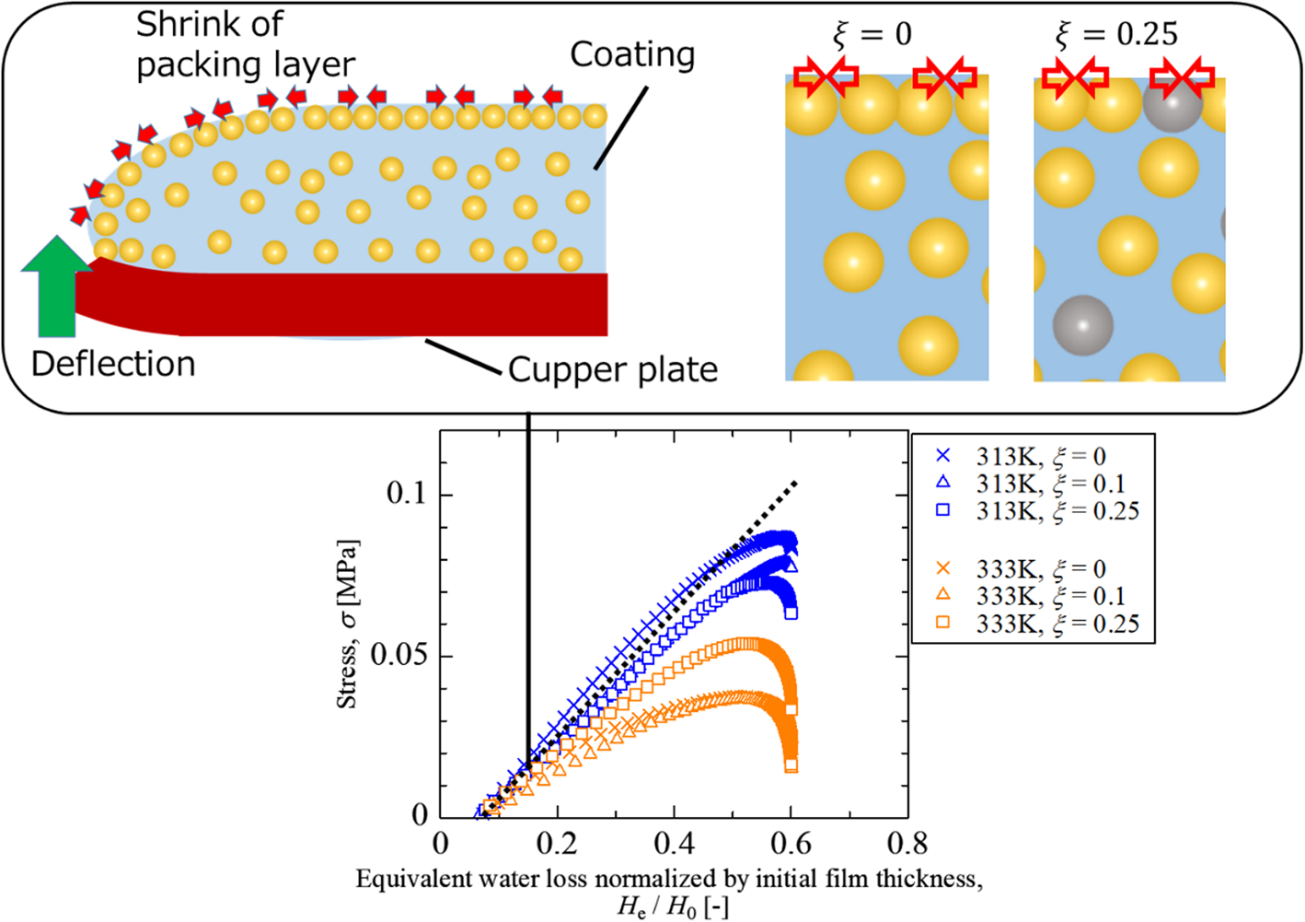
Stress histories as functions of the normalized thickness of water loss

We assumed that the different trend in the stress generation and relaxation between 313 and 333 K is caused by the accumulation and deformation of particles at the drying surface. Figure 8 shows the stress development for the normalized thickness of water loss. Note that the amount of packed layer is proportional to the thickness of water loss at Pe>>1. At 313 K, resin particles will behave as rigid spheres like silica particles in the early stage of the drying process. Since all particles, regardless of resin or silica, are accumulated at the drying interface without deformation, stress development in the former stage is proportional to the amount of packed layer at the surface, regardless of silica content. The stress development curve deviates from the linear relationship, expressed by a black dashed line, at ${H}_{\text{e}}/{H}_{0}=0.4.$ The volume fraction of nonvolatile components at the moment is roughly 70vol%, which indicates a random close-packing or the closest packing structure of particles is formed entirely. After that, the resin particle starts to be deformed by capillary deformation. Since the condensation of particle dispersion no longer contributes to increasing the deflection, the shrinkage of the particle packing layer with resin particle deformation dominates the stress. The stress stored in the packing layer is partially relaxed by the deformation of resin particles, so the stress is less than the proportional relationship.

At 333 K, a significantly large Péclet number and small $\overline{\lambda }$ indicate that rigid silica and deformable resin particles are simultaneously accumulated at the drying surface as described in Table 1. The linear stress-increasing trend in the early stage indicates that wet sintering is still not significant and that the accumulation layer of particles is thin enough not to affect the stress generation. As drying proceeds, wet sintering is dominant and the deformation of resin particles induces stress relaxation. As a result, the stress was smaller than the linear relationship.

Our previous study proposed a model for the development of deflection during the formation of the packing structure at the top for the latex-only coating layer. The model considers the reduction in elasticity of resin with the increase in sample temperature in the falling drying rate period as expressed by Eq. (12).

12 $$\Delta d={C}_{1}\times {\left(\frac{G\left({T}_{\text{m}}\right)}{{G}_{\text{C}}}\right)}^{\alpha }\times {H}_{\text{e}}$$

$G({T}_{\text{m}})$ and ${G}_{\text{C}}$ are elastic moduli at the temperatures of ${T}_{\text{m}}$ and ${T}_{\text{C}}$, respectively. Since resin particles do not deform and do not suppress the drying rate in the constant drying rate period, the term of $G\left({T}_{\text{m}}\right)/{G}_{C}$ is unity, expressing the linear relationship between deflection and equivalent thickness of water loss. The exponent $\alpha$ indicates the contribution of temperature change to resin deformability when wet sintering occurs. The previous study reported that $\alpha$ = 0.1 reasonably expresses the deflection behavior of the latex-only coating layer at 333 K. In silica–latex coating layers, only resin particles contribute to the stress relaxation and the elastic modulus of silica particles will not change. Therefore, by using the silica content $\xi$, the deflection in the silica–latex coating can be expressed by the following equation.

13 $$\Delta d={C}_{1}\times \left\{{\xi +\left(1-\xi \right)\left(\frac{G\left(T\right)}{{G}_{C}}\right)}^{\alpha }\right\}\times {H}_{\text{e}}$$

**Fig. 9:**
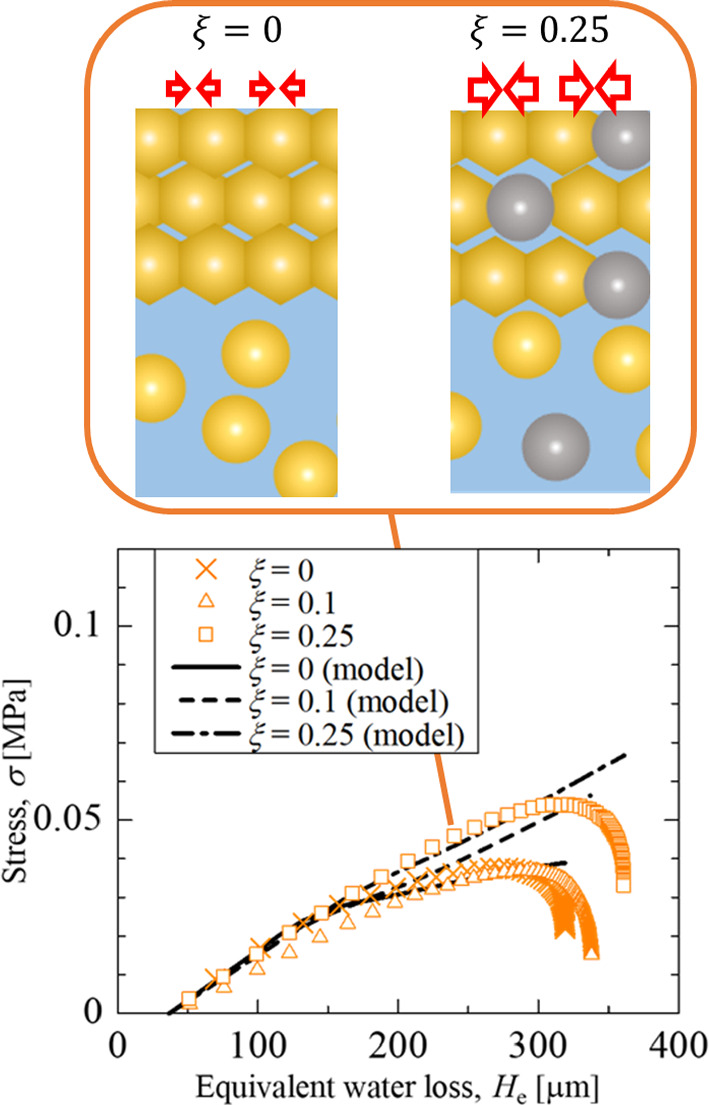
Stress histories as functions of the equivalent thickness of water loss at 333 K

Figure 9 shows the measured stress and model calculation results using predicted deflection as functions of the equivalent thickness of water loss. The black lines that appeared in Fig. 9 are the result of these model predictions using $\alpha$ = 0.1. It is found that the modified model reasonably explains the deflection behavior for the silica–latex coating layers at $\xi$ = 0.1 and 0.25. It was revealed that stress relaxation was caused only by the resin particles, and the amount of relaxation depended on the amount of resin deposited on the surface.

At the water loss of 250 µm, corresponding to the shrink ratio of roughly 40%, the measured stress deviates from the prediction and starts decreasing significantly. 40vol% latex coating layer converted to the random close-packing structure when the layer thickness decreased by 37vol%. Similarly, silica particles are uniformly dispersed in the closest packing structure of polymer particles when 40vol% silica–latex coating layer is shrunk by 43% at *ξ* = 0.25. When the surface layer of the close-packing structure of polymer particles reaches the bottom, the stress relaxation caused by polymer network deformation is a more primal factor than the stress generated due to the shrinking of the coating.

## Conclusion

We measured the drying rate and stress for silica–latex coating layers under convective drying conditions. The volume fraction of non-volatile components was constant at 40vol%, and the silica volume fraction in the non-volatile components was changed. The experiment clarified the effect of the packing structure formed by rigid and deformable particles on the film formation process.

It was found that the drying rate, with or without silica particles, is largely governed by the packing behavior of deformable resin particles. The drying rate change can be classified into the former and latter stages at the critical point, where the random close packing structure of the resin particles is formed throughout the coating layer. Before the critical point, the drying rate was nearly constant when accumulated resin particles are considered rigid, but the drying rate decreased significantly when resin particles were deformed by wet sintering. After the critical point, the drying rate is governed by the amount of residual water in the gap between deformed resin particles, regardless of drying temperature. In either drying stage, the drying rate could be explained by the structure formed by water and resin particles, as in the case of latex-only coatings, if the effect of silica content is properly considered.

On the other hand, it is found that stress generation is caused by the structures formed by resin and silica particles. Stress relaxation depends on the proportion of deformable resin particles and the temperature dependency of the elastic modulus of the resin. We then modified the simple model proposed for a latex-only coating to express the stress change for the latex–silica coatings.

This study provides insight into the drying of particle-containing latex coatings under more practical conditions. In future work, drying experiments of particle-latex coatings under various conditions will provide a more detailed estimation of the drying process.

## Data Availability

The datasets generated and analyzed during the current study are available from the corresponding author on reasonable request.

## List of symbols

*A*: Evaporation area, (m^2^)
*C*: Specific heat capacity, (J/(kg·K))
*d*: Deflection of copper substrate, (m)
*D*: Diffusion coefficient, (m^2^/s)
*E*: Young’s modulus, (N/m^2^)
G: Shear modulus, (N/m^2^)
*h*: Boundary heat transfer coefficient, ($\text{W}/({\text{m}}^{2}\text{ K}))$
*H*: Coating layer thickness, (m)
$H_{0}$: Initial coating film thickness, (m)
$H_{{\text{e}}}$: Equivalent thickness of water loss,(m)
$H_{{\text{r}}}$: Equivalent thickness of accumulated resin particles, (m)
$l$: Length for measuring substrate deflection, (m)
$L_{{\text{w}}}$: Latent heat of the evaporation of water, (J/kg)
$R_{{\text{w}}} { }$: Drying rate, (kg/m^2^ s)
*r*: Particle radius, (m)
*t*: Time, (s)
*T*: Temperature, (K)
$T_{{\text{C}}}$: Sample temperature during constant drying rate, (K)
$T_{{\text{g}}}$: Glass transition temperature, (K)
$u_{{\text{w}}}$: Moisture content, (m^3^ W/m^3^ NV)
$$v$$: Evaporation rate, (m/s)
$$V$$: Volume, (m^3^)
$$W$$: Mass, (kg)
$$Pe$$: Péclet number, (–)
$\overline{\lambda }$: Dimensionless time for indicating particle deformability, (–)
$\eta_{0}$: Zero-shear viscosity of resin, ($\text{Pa s}$)
$$\mu$$: Solvent viscosity, ($$\text{Pa s}$$)
$$\nu$$: Poisson’s ratio, (–)
$\xi$: Volume fraction of silica particle in NV, (–)
$$\rho$$: Density, (kg/m^3^)
$$\sigma$$: Stress, (Pa)
$\phi$: Particle volume fraction in sample, (–)

## Subscript

a: Hot air
b: Substrate
c: Coating
m: Sample
NV: Nonvolatile component
r: Resin particle
s: Silica particle
w: Water
